# Selective Cytotoxicity of Dihydroorotate Dehydrogenase Inhibitors to Human Cancer Cells Under Hypoxia and Nutrient-Deprived Conditions

**DOI:** 10.3389/fphar.2018.00997

**Published:** 2018-09-04

**Authors:** Yukiko Miyazaki, Daniel K. Inaoka, Tomoo Shiba, Hiroyuki Saimoto, Takaya Sakura, Eri Amalia, Yasutoshi Kido, Chika Sakai, Mari Nakamura, Anthony L. Moore, Shigeharu Harada, Kiyoshi Kita

**Affiliations:** ^1^School of Tropical Medicine and Global Health, Nagasaki University, Nagasaki, Japan; ^2^Department of Biomedical Chemistry, Graduate School of Medicine, The University of Tokyo, Tokyo, Japan; ^3^Department of Host-Defense Biochemistry, Institute of Tropical Medicine (NEKKEN), Nagasaki University, Nagasaki, Japan; ^4^Department of Applied Biology, Graduate School of Science and Technology, Kyoto Institute of Technology, Kyoto, Japan; ^5^Department of Chemistry and Biotechnology, Graduate School of Engineering, Tottori University, Tottori, Japan; ^6^Biochemistry and Medicine, School of Life Sciences, University of Sussex, Brighton, United Kingdom

**Keywords:** pyrimidine *de novo* biosynthesis, ubiquinone binding-site inhibitor, tumor bioenergetics, tumor microenvironment, structure–activity relationship, anticancer activity, ascofuranone, crystal structure

## Abstract

Human dihydroorotate dehydrogenase (HsDHODH) is a key enzyme of pyrimidine *de novo* biosynthesis pathway. It is located on the mitochondrial inner membrane and contributes to the respiratory chain by shuttling electrons to the ubiquinone pool. We have discovered ascofuranone (**1**), a natural compound produced by *Acremonium sclerotigenum*, and its derivatives are a potent class of HsDHODH inhibitors. We conducted a structure–activity relationship study and have identified functional groups of **1** that are essential for the inhibition of HsDHODH enzymatic activity. Furthermore, the binding mode of **1** and its derivatives to HsDHODH was demonstrated by co-crystallographic analysis and we show that these inhibitors bind at the ubiquinone binding site. In addition, the cytotoxicities of **1** and its potent derivatives **7**, **8**, and **9** were studied using human cultured cancer cells. Interestingly, they showed selective and strong cytotoxicity to cancer cells cultured under microenvironment (hypoxia and nutrient-deprived) conditions. The selectivity ratio of **8** under this microenvironment show the most potent inhibition which was over 1000-fold higher compared to that under normal culture condition. Our studies suggest that under microenvironment conditions, cancer cells heavily depend on the pyrimidine *de novo* biosynthesis pathway. We also provide the first evidence that **1** and its derivatives are potential lead candidates for drug development which target the HsDHODH of cancer cells living under a tumor microenvironment.

## Introduction

Mitochondria play important roles in energy metabolism by eukaryotic cells. The mammalian mitochondrial electron transport chain consists of four enzyme complexes located in the mitochondrial inner membrane: complexes I, II, III, and IV. Complexes I and II transfer reducing equivalents from NADH and succinate, respectively, to complex III *via* the ubiquinone pool, and complex III further transfers these equivalents to complex IV via cytochrome *c*. Electrons from complex IV are finally transferred to dioxygen, resulting in the production of water. ATP synthase produces ATPs by oxidative phosphorylation utilizing the transmembrane electrochemical gradient maintained by proton pumping activities of complexes I, III, and IV (Alberts, 2014). In addition to energy metabolism, mitochondria are also important organelle for pyrimidine *de novo* biosynthesis. In mammals, under physiological condition, pyrimidines are synthesized through both *de novo* biosynthesis and salvage pathways (Berg et al., 2015). Among the six enzymes from the *de novo* biosynthesis pathway, dihydroorotate dehydrogenase (DHODH), the fourth enzyme and rate-limiting step, catalyzes the electron transfer from dihydroorotate to the flavin mononucleotide (FMN) and from reduced FMN to an acceptor. Depending on its localization, DHODH can be classified as family 1 and 2. Family 1 DHODHs are cytosolic enzymes and further sub-classified as family 1A and 1B according to the ability to use fumarate (Inaoka et al., 2008; Kubota et al., 2018) or NAD^+^ (Jensen and Bjornberg, 1998) as electron acceptor, respectively. DHODHs from trypanosomatid parasites belong to family 1A and have been suggested to be drug targets to combat Chagas disease (Inaoka et al., 2016, 2017), African trypanosomiasis (Arakaki et al., 2008; Kubota et al., 2018), and leishmaniasis (Pinheiro et al., 2013). Family 2 DHODHs are membrane-bound enzymes and use quinone pool as its acceptor (Rawls et al., 2000) and thus, contribute to the formation of the electrochemical gradient through complexes III and IV activities (Löffler et al., 1997; Rawls et al., 2000). Hence, family 2 DHODH is the key enzyme linking pyrimidine *de novo* biosynthesis pathway and the respiratory chain. Indeed, it has been reported that inhibition of complex III impairs the efficiency of pyrimidine *de novo* biosynthesis (Khutornenko et al., 2010). Family 2 DHODHs are conserved in several pathogens such as *Helicobacter pylori* (Copeland et al., 2000; Ohishi et al., 2018), those causing fungal infections (Oliver et al., 2016; Wiederhold, 2017), *Toxoplasma gondii* (Hortua Triana et al., 2016), and *Plasmodium falciparum* (Singh et al., 2017). The ubiquinone binding site in *P. falciparum* DHODH (*Pf*DHODH) is known to be significantly divergent from its human counterpart and several groups have reported on the discovery of parasite-specific DHODH inhibitors (Booker et al., 2010; Wadood and Ulhaq, 2013; Xu et al., 2013; Kokkonda et al., 2016; Vyas et al., 2016; Azeredo et al., 2017; Maetani et al., 2017). Recently, Phase Ia/Ib studies of a potent and specific *Pf*DHODH inhibitor, DSM265, have been published with promising results (McCarthy et al., 2017; Sulyok et al., 2017). Intervenolin, a natural product isolated from *Nocardia* sp. ML96-86F2 (Kawada et al., 2013), and its derivatives were found to be potent inhibitors of *H. pylori* DHODH and proved to have greater efficacy for treatment of *H. pylori* infection compared to the conventional triple therapy (i.e., omeprazole, amoxicillin, and clarithromycin) (Ohishi et al., 2018). In human, it is known that pyrimidine *de novo* biosynthesis is upregulated in cancer and activated immune cells as well as virus-infected cells in order to meet the high demand for pyrimidines due to enhanced cell/virus proliferation compared to normal/uninfected cells (Ahmed, 1984; Weber et al., 1987; Okesli et al., 2017). Leflunomide, a human DHODH (HsDHODH) inhibitor currently used for the treatment of rheumatoid arthritis (RA), has been reported to have an anti-cancer and anti-viral activity (Teschner and Burst, 2010; Vyas and Ghate, 2011; Lolli et al., 2018). Leflunomide is a prodrug that is metabolized to teriflunomide (or A771726) and has a long half-life in blood, a property, however, which becomes a disadvantage once secondary infection appears. The high cost required for treatment of RA by leflunomide (Benucci et al., 2011) in addition to other disease-modifying antirheumatic drugs (i.e., cyclosporine A, sulfasalazine, leflunomide, and methotrexate) suggests that alternative HsDHODH inhibitors with lower treatment costs which are easier to administer are desired.

Ascofuranone (AF or compound **1**), a prenylphenolic compound produced by filamentous fungi, *Acremonium sclerotigenum* (Hijikawa et al., 2016), was reported to strongly inhibit the ubiquinol oxidase activity of *Trypanosoma brucei* mitochondrial alternative oxidase (TAO) (Minagawa et al., 1996; Kido et al., 2010), an enzyme essential for parasite survival which is absent in mammals (Shiba et al., 2013). Besides its anti-trypanosomal activity, **1** and its derivatives are known to have anti-cancer (Magae et al., 1986) and anti-viral activity (Tamura et al., 1968) in mammals. The target of **1** in mammals has previously been unidentified, but recent reports indicate that **1** is an HsDHODH inhibitor (Kita et al., 2012; Shen et al., 2016). It has been reported that several inhibitors of mitochondrial respiratory chain complexes such as rotenone (Complex I) and atpenin A5 (Complex II) exhibit specific cytotoxicity on pancreatic cancer cells only under tumor microenvironment mimicking conditions (such as hypoxia and nutrient-deprived culture) (Momose et al., 2010). Considering that DHODH is an important enzyme required for pyrimidine *de novo* biosynthesis and shuttles reducing equivalents to the mitochondrial respiratory chain, we have previously hypothesized that HsDHODH inhibitor may also show specific cytotoxicity to mammalian cells under microenvironment conditions, particularly when dioxygen and the substrate for pyrimidine salvage pathway are limiting (Tomitsuka et al., 2009; Sakai et al., 2012).

In this study, we have determined the inhibition mechanism of **1** and its functional groups essential for HsDHODH inhibition, through a comprehensive structure–activity relationship (SAR) study including co-crystal structures of HsDHODH with **1** and its derivatives. Furthermore, we have examined the anti-cancer effects of **1** on a panel of 39 types of human cancer cells. We show that **1** and its derivatives have little effect on the growth of DLD-1 cancer cells under normal culture conditions; however, under hypoxia and nutrient-deprived conditions, the survival rate of these cancer cells is drastically decreased. Our studies indicate that HsDHODH is a potential drug target and that **1** is a lead compound to develop new therapeutic agent targeting tumors living under microenvironment condition.

## Materials and Methods

### Protein Purification, Crystallization, and Enzyme Activity

Recombinant human DHODH was purified, crystallized, and assayed as previously described (Inaoka et al., 2016) without modification. Bovine mitochondrial fractions were prepared as previously described (Kita et al., 1988) and NADH dehydrogenase (complex I), succinate:quinone reductase (complex II), quinol oxidase (complexes III–IV), NADH-cytochrome *c* reductase (complexes I–III), and succinate-cytochrome *c* (complexes II–III) activities were assayed following an established method (Takamiya et al., 1986; Miyadera et al., 2001; Matsumoto et al., 2008; Kido et al., 2010; Nihashi et al., 2017).

### Synthesis of **1** and Its Derivatives

**1** and all of its derivatives (compounds **2**–**23**, see **Tables 1**–**3**) were synthesized essentially as previously described (Kita et al., 2012; Saimoto et al., 2012, 2015).

**Table 1:** Inhibition of human dihydroorotate dehydrogenase (DHODH) by furanone ring substituted ascofuranone (**1**) derivatives.

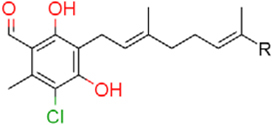

Compound	R	IC_50_ (nM)
Ascofuranone (**1**)	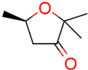	37.5 ± 12
Rac-AF (**2**)	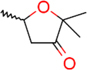	97.4 ± 7.3
CCB (**3**)	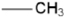	74.1 ± 22
**4**	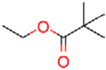	98.0 ± 5.5
**5**	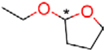	32.9 ± 2.7
**6**	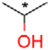	63.9 ± 11
**7**	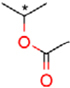	6.4 ± 0.2
**8**	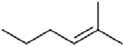	6.0 ± 0.5
**9**	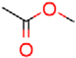	4.2 ± 0.4


*The IC_*50*_ of reference compounds brequinar and A771726 was 4.6 ± 0.5 and 773 ± 78 nM, respectively, in our assay condition described in the section “Materials and Methods.” Oxygen and chlorine atoms were colored in red and green, respectively, to facilitate the visualization of compounds in other figures generated by Pymol.*

**Table 2:** Inhibition of human DHODH by **1** derivatives with different linker structures.

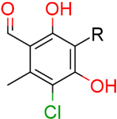

Compound	R	IC_50_ (nM)
Ascochlorin (**10**)	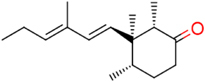	>1,000 (33%)
11	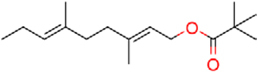	67.2 ± 2.8
12	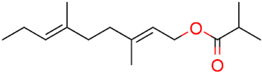	21.5 ± 0.3
13	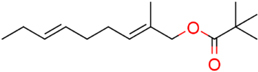	349 ± 96
14	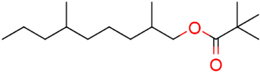	>1,000 (35%)
15	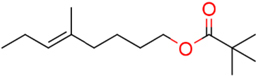	123 ± 18
16	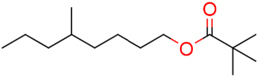	>1,000 (21%)
17	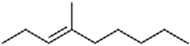	71.4 ± 2.4
18	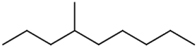	>1,000 (37%)


*Value in parenthesis represents the average inhibition (%) at 1,000 nM concentration of the derivatives. Oxygen and chlorine atoms were colored in red and green, respectively, to facilitate the visualization of compounds in other figures generated by Pymol.*

**Table 3:** Inhibition of human DHODH by **1** derivatives with different substitutions on benzene group.

Compound	Structure	IC_50_ (nM)
19	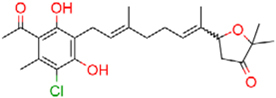	>1,000 (3%)
20	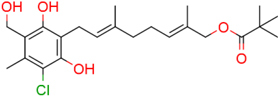	3,530 ± 0.51
21	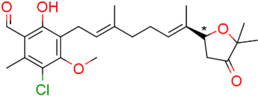	>1,000 (3%)
Colletorin B (22)	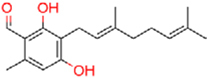	>1,000 (20%)
24	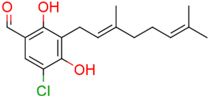	>1,000 (36%)


*Value in parenthesis represents the average inhibition (%) at 1,000 nM concentration of the derivatives. Oxygen and chlorine atoms were colored in red and green, respectively, to facilitate the visualization of compounds in other figures generated by Pymol.*

### Data Collection and Structure Determination

X-ray diffraction data of HsDHODH-**1** complex were collected at 100 K on the beamline BL41XU (λ = 1.00000 Å; Rayonix MX225HE CCD detector) at SPring-8 (Harima, Japan). X-ray diffraction data of compounds **6**, **7**, **8**, **9**, and **12** complex crystals were collected at 100 K on the beamline BL-17A (λ = 0.98000 Å; ADSC Quantum 315r) at Photon Factory (Tsukuba, Japan). For X-ray diffraction experiments at 100 K, a crystal mounted on a nylon loop was transferred to reservoir solution supplemented with 20% (w/v) glycerol and was then flash-frozen in liquid nitrogen stream. All data sets were processed and scaled using HKL-2000 (Otwinowski and Minor, 1997). The structure of the HsDHODH-**9** complex was solved by molecular replacement using the refined protein coordinates of HsDHODH-mii-4-087 complex (PDB code 3W7R) (Inaoka et al., 2016) as a search model. All other structures of HsDHODH inhibitor complexes were solved by molecular replacement using the refined protein coordinates of HsDHODH-**9** complex (PDB code 5ZF4) as a search model. MOLREP program (Vagin and Teplyakov, 1997) as implemented within CCP4 (Winn et al., 2011)^1^ was used for molecular replacement. Structural adjustments were made by iterative cycles of manual adjustments in COOT (Emsley and Cowtan, 2004) and refinements by REFMAC5 (Murshudov et al., 1997). Statistics of X-ray data collection and refinement are summarized in **Supplementary Table S1**. Graphical representations were generated with PyMOL^2^.

### 39-Cancer Cell Panel Assay

The growth inhibition activity of compounds **1**, **6**, **7**, **8**, **9**, and **12** against 39 types of human cancer cell lines was evaluated as previously reported (Yamori, 2003). This panel of human cancer cell lines consists of the following 39 human cancer cell lines: lung cancer, NCI-H23, NCI-H226, NCI-H522, NCI-H460, A549, DMS273, and DMS114; colorectal cancer, HCC-2998, KM-12, HT-29, HCT-15, and HCT-116; gastric cancer, MKN-1, MKN-7, MKN-28, MKN-45, MKN-74, and St-4; ovarian cancer, OVCAR-3, OVCAR-4, OVCAR-5, OVCAR-8, and SK-OV-3; breast cancer, BSY-1, HBC-4, HBC-5, MDA-MB-231, and MCF-7; renal cancer, RXF-631L and ACHN; melanoma, LOX-IMVI; glioma, U251, SF-295, SF-539, SF-268, SNB-75, and SNB-78; and prostate cancer, DU-145 and PC-3. All the above cancer cell lines from the panel were cultured in RPMI 1640 medium with 5% (v/v) fetal bovine serum, 100 units/ml penicillin, and 100 μg/ml streptomycin at 37°C under 5% CO_2_ atmosphere.

### Cell Culture

Human colorectal adenocarcinoma cells (DLD-1; Taiho Pharmaceutical Company, Japan) were grown in RPMI-1640 (Gibco), and human dermal fibroblast cells (HDF; Zenbio, Inc., United States) were grown in DMEM/F12 (Gibco), containing 10% heat-inactivated fetal bovine serum (FBS, Gibco) at 37°C under 5% CO_2_. For normal cell culture condition, cells were maintained under 5% CO_2_ and 21% oxygen. For hypoxia and nutrient-deprived culture conditions, cells were incubated under 1% oxygen in glucose and glutamine-free DMEM (Gibco) without FBS.

### Cytotoxicity Assay

DLD-1 or HDF cells were seeded at 2.5 × 10^4^ cells/well on a 96-well plate with normal medium, and cultured overnight under normal culture condition. The cells were washed with PBS and the medium was replaced to either normal medium or glucose and glutamine-free DMEM without FBS. Test compounds or DMSO as a control were added to the wells. The cells in normal medium were cultured under normal culture conditions, while cells in nutrient-deprived medium were cultured under hypoxia condition. After 24 or 48 h incubation, the cells were washed with PBS, 100 μl of fresh normal medium, and 10 μl of Cell Counting Kit-8 solution (Donjindo, Japan) was added to each well. After 3 h incubation under normal culture condition, the absorbance was measured at 450 nm using SpectraMax M2e-TUY microplate reader (Molecular Devices). Cell viability of test wells was calculated based on absorbance of control wells containing DMSO according to manufacturer’s protocol.

## Results

### Structure–Activity Relationship of **1** Against HsDHODH

**FIGURE 1:**
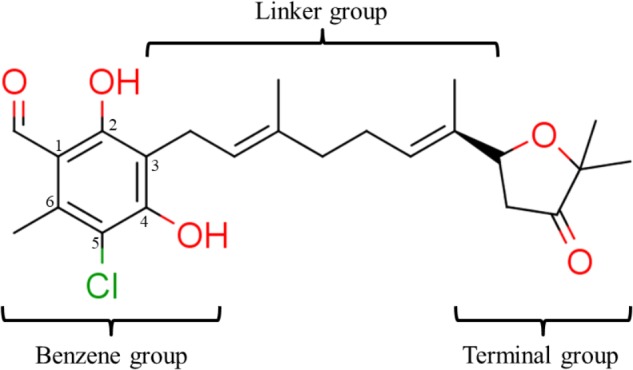
Structure of ascofuranone (AF or **1**). The benzene, linker and terminal groups are indicated. Oxygen and chlorine atoms were colored in red and green, respectively, to facilitate the visualization of compounds in other figures generated by Pymol.

We have previously designed and synthesized several **1** derivatives targeting TAO for anti-trypanosomal drug development (Saimoto et al., 2012; Shiba et al., 2013). After evaluation of over 100 derivatives of **1** against HsDHODH activity, a comprehensive SAR study was conducted. As shown in **Figure 1**, the structure of **1** can be divided into benzene, linker and terminal groups. Initially, several **1** derivatives possessing changes in the terminal group were evaluated (**Table 1**). Under our assay condition, the reference compounds brequinar and A771726 inhibited HsDHODH with IC_50_ of 4.6 and 773 nM, respectively, while **1** inhibited HsDHODH with an IC_50_ of 37.5 nM (**Table 1**). When racemic-AF (**2**) was tested, the IC_50_ increased to 97.4 nM, indicating that the *S*-isomer (1) is preferable for inhibition of HsDHODH. The terminal group from **1** (furanone ring) was shown to be dispensable for HsDHODH inhibition, as indicated by the IC_50_ of colletochlorin B (**3**) (74.1 nM). Consistent with this notion, several groups replacing the furanone ring from **1**, such as **4**, **5**, and **6**, have little effect on the IC_50_s as shown in **Table 1**. However, changing the furanone ring to other groups such as in **7**, **8**, and **9**, showed a sharp decrease in the IC_50_s to 6.4, 6.0, and 4.2 nM, respectively. This indicates that, although the furanone ring is not required for HsDHODH inhibition, its terminal group should be explored in an attempt to obtain derivatives with higher potency than **1**. Secondly, modifications at the linker group were also evaluated (**Table 2**). Ascochlorin (**10**), another metabolite isolated from *A. sclerotigenum* which has a shorter linker length than **1**, has previously been reported to potently inhibit complex III by binding at both Q_o_ and Q_i_ sites (Berry et al., 2010). However, **10** was not an effective HsDHODH inhibitor and even at 1,000 nM it only inhibited HsDHODH activity by 33% (**Table 2**). A comparison of **4** and **11** indicates that a change in the methyl group position from the second isoprene unit does not affect their inhibition potency (IC_50_ of 98.0 nM versus 67.2 nM, respectively). However, when the terminal pivaloyl group from **11** was changed to an isopropyl group **12** (IC_50_ of 21.5 nM), there was about threefold increase in inhibition potency. Compared to **4**, where the methyl group from the first isoprene unit was removed (see **13**), the IC_50_ increased to 349 nM (about 3.5-fold decrease), indicating that this group may play a significant role in the binding of the inhibitor (**Table 2**). Of particular interest was the finding, as shown by compound **14**, that when all of the double bonds from the linker group are removed, inhibition activity is abolished. Hence, it was important to evaluate the individual contribution of the double bonds in linker group (**Table 2**). It became evident that the double bond at the first isoprene unit is the one critical for HsDHODH inhibition (**Table 2**), as shown by the change in the IC_50_s of **15** (123 nM)/**16** (>1,000 nM) and **17** (71.4 nM)/**18** (>1,000 nM) pair compounds. Finally, the essential groups from benzene group were evaluated (**Table 3**). When the 1-aldehyde group from **2** was changed to a ketone group **19**, the inhibition activity was lost. Similarly, reduction of 1-aldehyde to hydroxyl group in **20** increased the IC_50_ to 3,530 nM. Next, the contribution of 4-O^-^ group was evaluated. Comparison of **1** and **21** makes it clear that the 4-O^-^ group is indeed essential for inhibition. Removal of 5-chlorine in **22** as well as the 6-methyl in **23** also showed >13-fold increase in the IC_50_s in comparison to **3**. As indicated in **Table 3**, any substitution at positions 1, 4, 5, and 6 in the benzene ring (**Figure 1**) have a negative impact on HsDHODH inhibition activities. The contribution of 2-OH group to inhibition of HsDHODH could not be assessed in this study due to difficulties in the synthesis of **1** derivatives with substitutions only at this position in the benzene ring.

### Structural Insights Into the HsDHODH Inhibition Mechanism by **1** and Its Derivatives

**FIGURE 2:**
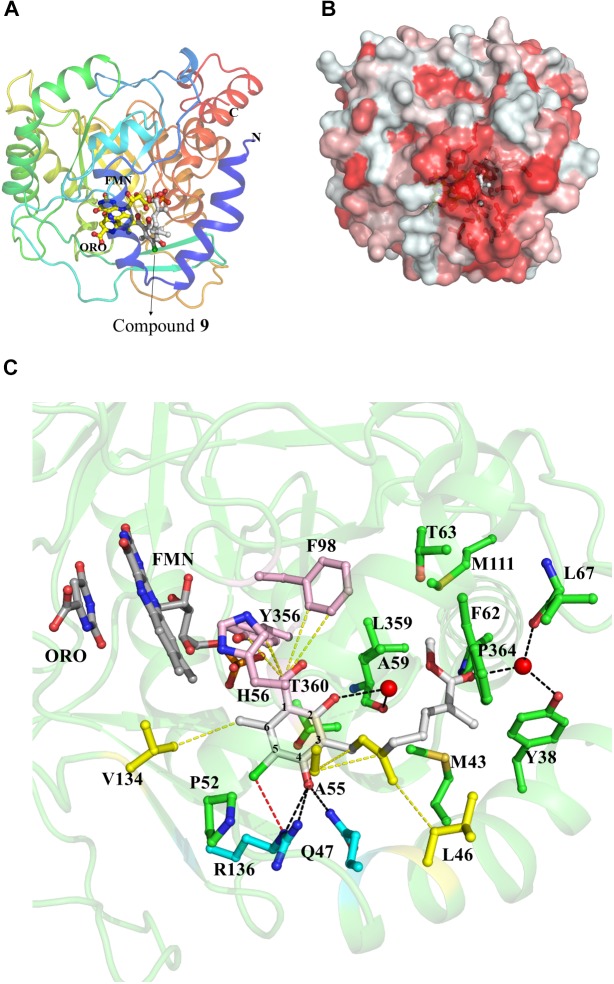
Representative crystal structure of HsDHODH in complex with **1** and its derivatives. **(A)** Overall structure of HsDHODH in complex with **9**. Cartoon model is colored in rainbow from blue (N terminus) to red (C terminus). FMN and orotate (ORO) molecules are shown in yellow ball and stick models. Compound **9** is shown in white ball and stick model. **(B)** Surface presentation of HsDHODH in complex with **9** view from the same angle as **(A)**. Colors are according to the following hydrophobicity scale: red, high hydrophobicity and white, low hydrophobicity (www.pymolwiki.org/index.php/Color_h). **(C)** Inhibitor binding site of HsDHODH. FMN and orotate (ORO) molecules are shown in gray ball and stick models. Compound **9** is shown in white ball and stick model. The black dashed lines represent hydrogen bonds. The yellow dashed lines represent important hydrophobic interactions. The red dashed line represents halogen bond. Water molecules, which interact with both **9** and HsDHODH through hydrogen bonds, are shown as red sphere.

The binding mode of **1** and its derivatives was investigated by co-crystallographic analysis and through the generation of structures of HsDHODH in complex with **1**, **6**, **7**, **8**, **9**, and **12** (**Supplementary Figures S1**–**S6**, respectively). In all co-crystal structures, the inhibitors were bound at a hydrophobic cavity formed by N-terminal residues, which is believed to be the ubiquinone binding site (**Figures 2A,B**). All the substituents from the benzene group, that were found to be essential for inhibition of HsDHODH activity by the SAR analysis, interacted tightly with surrounding amino acid residues (**Figure 2C**). The 1-aldehyde group was found to interact with His56, Phe98, and Tyr356 through hydrophobic interaction ranging from 3.18 to 3.49 Å (**Figure 2C**). The 4-OH group, which was previously shown to be deprotonated (4-O^-^) at physiological pH (Berry et al., 2010), was within hydrogen bond distance to εN^2^ from Gln47 and εN/ηN^2^ from Arg136 (**Figure 2C**). The 5-chlorine and 6-methyl groups interact with εN from Arg136 through halogen bond and γC from Val134 through hydrophobic interactions, respectively (**Figure 2C**). An additional interaction between benzene 2-OH group and main chain carbonyl group from Leu359 which was mediated by a water molecule (**Supplementary Figure S5**) was only observed in compound **9**. This is a specific feature that could explain why compound **9** shows the most potent inhibition of HsDHODH activity. The double bond from the first isoprene unit, which was shown to be essential by the SAR analysis (**Table 2**), was found to interact with βC from Ala55 *via* CH—π bond (**Figure 2C**). Also, the methyl group can be seen to interact hydrophobically with βC from Leu46 (**Figure 2C**). The interaction of terminal group varied according to each inhibitor. For all compounds co-crystallized with HsDHODH in this study, the interaction of the terminal group and Met43, Thr63, and Pro364 were observed (**Supplementary Figures S1**–**S6**). Additional interaction is as follows: compounds **1** (**Supplementary Figure S1**), **7** (**Supplementary Figure S3**), and **12** (**Supplementary Figure S6**) with Leu359 through hydrophobic interactions; **6** showed an intermolecular hydrogen bond between terminal OH and benzene 2-OH bridged by a water molecule (**Supplementary Figure S2**); **8** with Leu359 and Met111 (**Supplementary Figure S4**); and **9** with Tyr38 (through a water molecule), Thr63, Leu67, Met111, and L359 (**Supplementary Figure S5**).

### Anti-proliferative Effect of **1** and Its Derivatives

Since HsDHODH is an anti-cancer drug target, the anti-proliferative effect of **1** and several derivatives was evaluated by subjecting them to a panel of 39 types of cancer cells from various origins developed at Japanese Foundation for Cancer Research (Yamori, 2003). Based on HsDHODH inhibition by **1** and its derivatives, the following compounds were selected for further analysis; the three other most potent derivatives (**7**, **8**, and **9**), including one which was more (**12**) and which is less (**6**) potent than **1** (**Table 4**). We used the COMPARE algorithm (Kong and Yamori, 2012) on a panel of human cancer cell lines to predict the molecular targets or evaluate the mechanism of action of test compounds through comparison of their growth inhibition profiles to standard anti-cancer drugs and chemical tools with known mechanism. The *r*-value obtained from COMPARE is used to estimate the degree of similarity between the test and standard compound pair. The *r*-value > 0.8 suggests similar mechanism of action between two compounds. In the case of **1** and its derivatives, the highest *r*-value ranged from 0.557 to 0.619, thus showing that **1** and its derivatives have no similarity to standard compounds tested and consequently indicate a new mechanism of action. The three most potent HsDHODH inhibitors (**7**, **8**, and **9**) tended to be more active while the least active (**12**) was also less active against 39 cancer cells from the panel (**Table 4**).

**Table 4:** Effect of **1** and its derivatives on 39 types of human cancer cells.

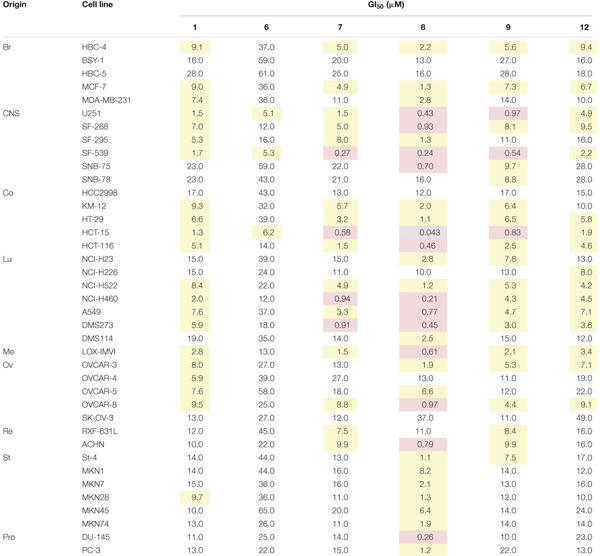

*The origin of cancer cell lines is as follows. Br, Breast; CNS, central nervous system; Co, colon; Lu, lung; Me, melanoma; Ov, ovary; Re, renal; St, stomach; Pro, prostate. Concentrations of **1** and its derivatives causing 50% growth inhibition (GI_*50*_) between 10 and 1 μM are colored in yellow, between 1 and 0.1 μM are colored in red and below 0.1 μM is colored in purple.*

### **1** and Its Derivatives Potently Inhibit the Viability of Human Cells Cultured Only Under Hypoxia and Nutrient-Deprived Conditions

**FIGURE 3:**
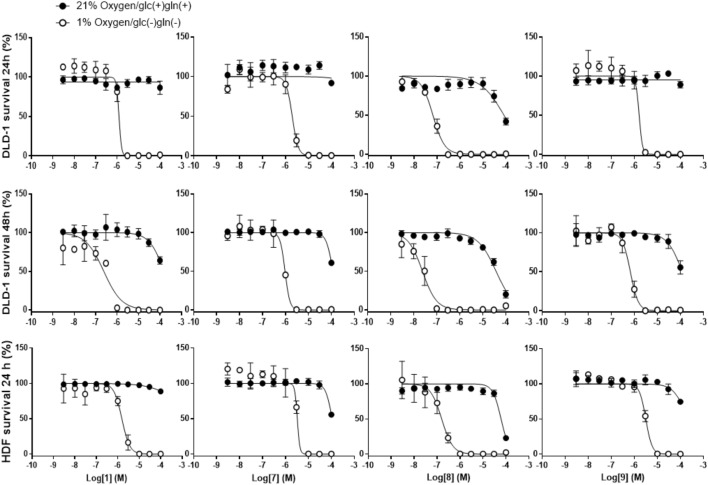
Effects of **1** and its derivatives on survival of DLD-1 and HDF cells under normoxia/nutrient-rich conditions (circles) and hypoxia and nutrient-deprived conditions (square). (Top panels) Survival rates of DLD-1 cells in presence of **1** and its derivatives for 24 h were measured using WST-8 assay. (Middle panels) Similar experiment was performed, however, increasing the incubation time with HsDHODH inhibitors to 48 h. (Bottom panels) Same experiment as in top panels was conducted using HDF cells. Each lane of panels, from left to right, shows the data obtained by incubation of indicated cells with **1**, **7**, **8**, and **9**, respectively. All data represent the average from three independent experiments.

Compound **10** (**Table 2**) is structurally related to **1** (**Figure 1**), it is a potent inhibitor of complex III and the first reported compound to bind at both Q_o_ and Q_i_ sites (Berry et al., 2010). To investigate the possibility of complex III inhibition, **1** and its derivatives were assayed against succinate-cytochrome *c* (complexes II–III) activity using bovine heart mitochondria (**Table 5**). Compared to IC_50_ of 13 nM for compound **10** against mammalian complex III (Berry et al., 2010), **1** and its derivatives have much higher IC_50_s, i.e., in the micromolar range, except for **8** which gave an IC_50_ of 120 nM (**Table 5**). Several other inhibitors of mitochondrial respiratory chain enzymes show preferential cytotoxicity to human pancreatic cancer cells (Panc-1) under hypoxia and nutrient-deprived conditions (Momose et al., 2010). To investigate whether **1** also has similar effects, we measured the viability of DLD-1 cells, which is derived from colon cancer and known to growth under tumor microenvironment, after **1** treatment under normal cell culture conditions or under hypoxia and nutrient-deprived conditions (**Table 5**). The viability of cells cultured in the original medium under normal oxygen concentration was approximately 80% even after exposure to 100 μM of **1** for 24 h (**Figure 3**, top panel). On the other hand, **1** showed remarkable reduction in the viability of cells cultured under hypoxia and nutrient-deprived conditions (**Figure 3**, top panel) with an IC_50_ value of 1.2 μM (**Table 5**).

**Table 5:** IC_50_ values of **1** and its derivatives on cultured cancerous (DLD-1) and non-cancerous (HDF) human cells at 24 or 48 h.

Compounds	IC_50_ (μM)					
	
	DLD-1 (24 h)		DLD-1 (48 h)		HDF (24 h)	
	
	Normoxia nutrient (+)	Hypoxia nutrient (-)	Normoxia nutrient (+)	Hypoxia nutrient (-)	Normoxia nutrient (+)	Hypoxia nutrient (-)
1	>100	1.2 ± 0.79	>100	0.24 ± 0.057	>100	1.5 ± 0.18
7	>100	2.0 ± 0.19	>100	0.95 ± 0.059	>100	3.4 ± 0.36
8	81 ± 24	0.066 ± 0.042	38 ± 3.3	0.024 ± 0.0030	61 ± 5.3	0.16 ± 0.023
9	>100	1.6 ± 0.42	>100	0.67 ± 0.066	>100	3.1 ± 0.19


*IC_*50*_ values were analyzed and calculated by GraphPad PRISM 6 (GraphPad Co. Ltd., United States).*

Next, the effect in the viability of the three most potent HsDHODH inhibitor **7**, **8**, and **9** was investigated. Similarly to **1**, all three derivatives exhibited strong reduction in viability specifically to the cells cultured under hypoxia and nutrient-deprived culture conditions (**Figure 3**, top panel and **Table 5**). Among the four compounds tested, **8** exhibited the most potent reduction in DLD-1 viability with IC_50_ value 20-fold lower (0.066 μM) than **1** (1.2 μM) (**Table 5**). Lower cell viability was also observed at a higher concentration of **8** under normal culture conditions (**Figure 3**, top panel and **Table 5**). Such a tendency was consistent with the result of the 39 cancer cell panel assay (**Table 4**). In all compounds tested, the reduction in the viability was even more pronounced after 48 h treatment compared to 24 h treatment (**Figure 3**, middle panel and **Table 5**). In order to evaluate the effect of **1** and its derivatives on non-cancerous cells, the viability of HDF cells after 24 h in the presence of compounds was also evaluated under normal condition in addition to hypoxia and nutrient-deprived conditions. As shown in **Figure 3** (bottom panel) and **Table 5**, the viability curve and the IC_50_s determined for all four compounds against HDF were very similar to the results obtained with DLD-1. Our results suggest that **1** and its derivatives are selectively reducing the viability of human cells cultured under hypoxia and nutrient-deprived conditions, regardless of cell type.

### At High Concentrations, **1** and Its Derivatives Inhibit the Activity of Respiratory Complex III

Since **1** and its derivatives inhibit HsDHODH by binding at the ubiquinone binding site, the inhibitory effect of **1**, **7**, **8**, and **9** against the mammalian respiratory complexes was investigated. As shown in **Table 6**, the mammalian complexes I–III (NADH cytochrome *c* reductase) activity was inhibited at micromolar to low micromolar order, except for **8** which show IC_50_ of 48 nM. The complexes II–III (succinate cytochrome *c* reductase) activity was also inhibited at slightly higher concentration than the IC_50_ of complexes I–III (**Table 6**). Next, the inhibition against individual respiratory complex activities were determined (**Table 6**). At 5 μM concentration, **1**, **7**, and **9** were poor inhibitors, while **8** inhibited 43% of complex I activity. None of the inhibitors tested significantly inhibited the complex II activity (**Table 6**). Finally, the ubiquinol oxidase activity of complex III was inhibited by **7**, **8**, and **9**. As positive control, rotenone, atpenin A5, and ascochlorin (**10**) were used for complexes I, II, and III, respectively (**Table 6**). Those results indicate that despite of potent HsDHODH inhibition, those compounds start to inhibit complex III activity at micromolar concentration.

**Table 6:** IC_50_ values of **1** and its derivatives on recombinant human DHODH and bovine complexes I–III, II–III, I, II, and III activities.

Compounds	IC_50_ (μM)			Inhibition (%)		
		
	HsDHODH	Complexes I–III	Complex II–III	Complex I	Complex II	Complex III
1	0.038 ± 0.012	1.5 ± 0.04	13 ± 1.7	11 ± 5.8	1.5 ± 7.6	36 ± 1.2
7	0.0065 ± 0.0002	0.46 ± 0.01	2.3 ± 0.16	15 ± 2.3	3.2 ± 6.7	98 ± 4.4
8	0.006 ± 0.0005	0.048 ± 0.001	0.12 ± 0.013	43 ± 1.8	8.2 ± 4.9	87 ± 2.8
9	0.0042 ± 0.0004	1.0 ± 0.02	5.7 ± 0.37	7.6 ± 4.2	5.3 ± 4.1	87 ± 0.9
Rotenone	>10	0.0063 ± 0.0001	>10	94 ± 1.5	6.4 ± 7.9	1.1 ± 2.8
Atpenin A5	>10	2.8 ± 0.44	0.0063 ± 0.0002	10 ± 2.7	97 ± 1.0	0.0 ± 4.6
10	2.3 ± 1.9	0.0033 ± 0.0001	0.0069 ± 0.0002	6.8 ± 4.1	3.6 ± 8.5	94 ± 2.4


*The effect of each inhibitors on individual respiratory complexes were determined at 5 μM concentration. Rotenone (1 μM), atpenin A5 (0.1 μM), and ascochlorin (**10**, 1 μM) were used as positive control for inhibition of complexes I, II, and III activities, respectively. IC_*50*_ values were analyzed and calculated by GraphPad PRISM 6 (GraphPad Co. Ltd., United States).*

## Discussion

In this study, we report on the first comprehensive SAR combined with co-crystal structures of **1** and its derivatives on HsDHODH. Interestingly, **1** was originally identified as having antitumor and antiviral activities (Sasaki et al., 1973a,b). Recently, at concentrations greater than 30 μM, **1** was reported to have anti-inflammatory activity by suppressing the expression of p-ERK1/2 and activation of NF-κB, AP-1(p-c-Jun) in RAW 264.7 macrophages (Park et al., 2017). At such high concentrations of **1**, not only is the activity of HsDHODH inhibited but also that of complex III activity and thus, it is not clear whether the anti-inflammatory response is the primary or a secondary effect following inhibition of respiratory chain enzymes. **1** and **10** are specific and potent inhibitors of HsDHODH and complex III, respectively, and care must be taken to interpret the biological data when using those two compounds.

The SAR studies of **1** were clear and the results can be summarized as follows: (i) the 1-aldehyde, 4-O^-^, 5-chlorine, and 6-methyl group are essential for HsDHODH inhibitory activity, (ii) the first isoprene unit from the linker is also critical for enzyme inhibition, and (iii) the second isoprene unit and the furanone ring are dispensable, however, depending on the substitutions at terminal group, the inhibition activity can increase. In our assay condition, the amount of purified enzyme used was fixed at 10 nM. Considering that the IC_50_s of **7**, **8**, and **9** were being close to 5 nM, we can conclude that these three derivatives bind to the HsDHODH at equimolar concentration (i.e., 1:1 binding).

In order to evaluate the anti-cancer activity, a panel of 39 human cancer cells was assayed in the presence of **1** and its derivatives. The results indicate that all HsDHODH inhibitors tested had anti-cancer activity. Among the compounds tested, compound **8** showed the strongest anti-cancer activity. This can be related to the ability of compound **8** to inhibit complexes I and III, in addition to HsDHODH, activities. Inhibition of complex III in cancer cells can have pleiotropic effect, since the electron flux from all other pathways, upstream of complex III, would be suppressed. In addition to the 39 cancer cell lines from the panel, we have also tested DLD-1 and non-cancerous cell HDF cells. DLD-1 was chosen because it was derived from colon cancer, which is known to growth under tumor microenvironment. In addition, those two cell lines were insensitive to high concentrations of **1** even after 48 h.

Tumor microenvironment is characterized by incomplete vascularization, thus, resulting in low oxygenation (hypoxia) and nutrient-deprived environment. The central regions of solid tumors in microenvironments are exposed to limited oxygen and nutrients because of vascular insufficiency (Brown and Giaccia, 1998). It was reported that even head and neck cancer, which is surrounded by relatively higher concentrations of oxygen, is exposed to only a quarter of normal oxygen tensions (Brown and Wilson, 2004). However, some anti-cancer drugs for clinical use, including 5-fluorouracil and bleomycin, show little cytotoxic effects on cancer cells under hypoxia and nutrient-deprived conditions (Teicher et al., 1981; Lu et al., 2004). In addition, they show low specificity for cancer cells, leading to severe side effects (Khan et al., 2012). Since the supply of pyrimidine precursors used for salvage pathway in the tumor microenvironment is limited, the cells need to upregulate *de novo* biosynthesis to meet the cellular pyrimidine demand. Accordingly, we have hypothesized that under the tumor microenvironment cells become highly dependent on the pyrimidine *de novo* pathways and are also hypersensitive to HsDHODH inhibitors. Colorectal cancer is such a kind of cells living under microenvironment conditions and we chose DLD-1 because of its resistance to **1** when compared to other cancer cells from the panel. This hypothesis is supported by the fact that **1** and the three most potent derivatives showed selective and potent cytotoxicity to DLD-1 cells under hypoxia and nutrient-deprived conditions. Our result also suggests that cells under normal conditions can survive using salvage pathway even in the presence of the DHODH inhibitors.

The co-crystal structures reveal that **1** and its derivatives inhibit HsDHODH by binding in the hydrophobic pocket formed by the N-terminal extension specific for family 2 enzymes, which we believed to be the ubiquinone-binding site. All substituents from benzene ring were completely surrounded by amino acid residues and interacting through hydrogen bonding, halogen bonds, and hydrophobic interactions with distances less than 4 Å. Co-crystal structures also revealed the hydrophobic interactions formed by the first isoprene unit which we have shown to be essential for inhibition. The interaction formed by the terminal group varied between inhibitors and, in general, the terminal group of the most potent inhibitors tended to exhibit more interactions than weaker ones. Accordingly, the binding mode of **1** and its derivatives revealed by the co-crystal structures correlate well with result obtained by our SAR study.

It has been reported that the activity of pyrimidine *de novo* biosynthesis was suppressed by the inhibition of mitochondrial complex III due to reduced turnover of ubiquinone necessary for DHODH activity (Khutornenko et al., 2010). Therefore, the rate of pyrimidine *de novo* biosynthesis in cells under hypoxia is expected to decrease due to reduced electron flux to dioxygen. In addition, it is likely that salvage pathways are also affected under tumor microenvironment conditions due to limited supply of pyrimidine precursors. Thus, it is still unclear how cells living under microenvironments such as solid tumors obtain pyrimidines to survive. We have previously demonstrated that when DLD-1 cells are cultured under hypoxia and nutrient-deprived conditions the Complexes III and IV activities are repressed while fumarate reductase (FRD) activity of mitochondrial complex II increases, which is the reverse reaction of complex II, succinate–ubiquinone reductase (SQR) (Tomitsuka et al., 2009). Under these conditions, the FRD activity of complex II functions as a terminal quinol oxidase (fumarate respiration) and facilitates the re-oxidation of NADH by complex I (NADH-FRD system), the generation of an electrochemical gradient and ATP synthesis (Tomitsuka et al., 2010). The advantage of FRD activity is its ability to re-route electron flux from respiratory quinone-dependent processes, including HsDHODH, to fumarate when dioxygen availability is low. The end-product of fumarate respiration is succinate, which accumulates in the extracellular compartment. Such an accumulation of succinate has been reported for cancer cells cultured under hypoxia and in mouse models of ischemia (Chouchani et al., 2014; Vazquez et al., 2016). We propose in this paper that under hypoxia and nutrient-deprived conditions, mitochondrial HsDHODH activity is connected to fumarate via a low redox potential quinone (probably menaquinone) (Vos et al., 2012) and complex II. In fact, this hypothesis is supported by a previous report showing that DHODH is physically associated with complex II in mitochondrial inner membrane (Fang et al., 2012) which would be advantageous for efficient electron flux from dihydroorotate to fumarate. In addition, in cancer cells cultured under hypoxia and nutrient-deprived conditions, Atpenin A5, which is a potent and specific complex II inhibitor, showed the strongest cytotoxic effects compared to other mitochondrial respiratory chain inhibitors (Momose et al., 2010). In such circumstances where the oxygen uptake is already limited, inhibition of fumarate respiration by Atpenin A5 must have pleiotropic effect. Since **1** and its derivatives are ubiquinone binding site inhibitors, enzymes from mammalian respiratory chain that use ubiquinone or ubiquinol such as complexes I, II, and III can potentially be inhibited. In order to verify this hypothesis, bovine sub-mitochondrial particles were used and the effect of **1**, **7**, **8**, and **9** on complexes I–III, II–III, I, II, and III activities was evaluated (**Table 6**). According to the biochemical assays, those compounds at 5 μM concentration showed no or little inhibition over complexes I and II while **7**, **8**, and **9** inhibited the activity of complex III over 80%, which is consistent to the observation where complexes I–III and II–III are also inhibited (**Table 6**). The inhibition of complex III may become relevant under normoxic condition where the electrons from ubiquinol pool flow to dioxygen via complexes III and IV. Because the activities of complexes III and IV are repressed or not functional under hypoxic condition (Tomitsuka et al., 2009), inhibition of complexes I or II rather than complex III can cause negative impact to the viability of cells. This is also supported by the potent growth inhibition activity of **8** under normoxia/nutrient-rich and hypoxia/nutrient-deprived conditions. Taking together, we can conclude that potent inhibition of cell viability specifically under hypoxia/nutrient-deprived condition is caused due to (i) inhibition of HsDHODH by **1**, **7**, and **9** and (ii) inhibition of both of HsDHODH and complex I by **8**. Under normoxia/nutrient-rich condition, **1** and its derivatives may exert antiproliferative effect due to additional inhibition of complex III. To the best of our knowledge, this is the first report on the discovery of HsDHODH equimolar binding inhibitors and evaluation of their anticancer activity under tumor microenvironment-mimicking condition.

We have also tested the effect of A771726, which is an HsDHODH inhibitor (IC_50_ = 773 ± 78 nM) with different scaffold used for treatment of RA, on DLD-1 under hypoxia/nutrient-deprived conditions. Because of lower potency of A771726 against HsDHODH than 1 and its derivatives, similar shift in the potency over DLD-1 was expected. Consistently, similar pattern of specific inhibition under hypoxia/nutrient-deprived condition was observed, however, at higher concentrations (100 μM) of A771726(**Supplementary Figure S7**). Although this study strongly suggest the potential to target HsDHODH in cancer cells living under tumor microenvironment, future studies will explore whether HsDHODH inhibitors with distinct scaffolds also exhibit hypoxia-selective anticancer activity.

Another significant finding is that the hypersensitivity to HsDHODH inhibitors was not limited to cancerous cells, as similar result was also obtained with HDF cells. These results indicate that under hypoxia and nutrient-deprived conditions, cancerous and non-cancerous cells are highly dependent on pyrimidine *de novo* biosynthesis. In addition, we have previously found FRD activity in a variety of cancer cells and also in HDF (Tomitsuka et al., 2009). At this point in time, it is tempting to speculate that the ability to switch from oxygen respiration to fumarate respiration in order to maintain the electrochemical gradient and pyrimidine *de novo* biosynthesis through complex I and DHODH, respectively, can be an important survival response conserved in other human cells. Further study is needed to elucidate the mitochondrial processes supported by FRD activity from complex II under tumor microenvironment and a chemogenomic validation of those pathways as drug target, including HsDHODH.

## Author Contributions

KK directed the work. HS, SH, and KK conceived the projects. YM, CS, and MN performed the cell assays. DI, ToS, EA, and YK purified, crystallized, and determined the structures of HsDHODH. DI, YK, and AM analyzed the SAR. HS synthesized the compound **1** and its derivatives. DI, TaS, and EA performed the inhibition studies. ToS performed the refinement of co-crystal structures and PDB depositions. YM, DI, ToS, and HS wrote the manuscript with comments from all authors. AM edited the manuscript.

## Conflict of Interest Statement

The authors declare that the research was conducted in the absence of any commercial or financial relationships that could be construed as a potential conflict of interest.

## Footnotes

1 http://www.ccp4.ac.uk/

2 http://www.pymol.org
