# Supplementary material for: Selective Cytotoxicity of Dihydroorotate Dehydrogenase Inhibitors to Human Cancer Cells Under Hypoxia and Nutrient-Deprived Conditions
Source: Front Pharmacol. 2018 Sep 4;9:997. doi: 10.3389/fphar.2018.00997 (PMC6131557; doi:10.3389/fphar.2018.00997)
Supplement: Supplementary file 1 [file Table_1.DOCX]

**Table S1. Data Collection and Refinement Statistics.**

|  | **1** | **6** | **7** | **8** | **9** | **12** |
| --- | --- | --- | --- | --- | --- | --- |
| **Data collection** |  |  |  |  |  |  |
| Beam line | BL41XU^b^ | BL17A^a^ | BL17A^a^ | BL17A^a^ | BL17A^a^ | BL17A^a^ |
| Space group | *P*3_2_21 | *P*3_2_21 | *P*3_2_21 | *P*3_2_21 | *P*3_2_21 | *P*3_2_21 |
| Cell dimensions |  |  |  |  |  |  |
| *a, c* (Å) | 90.3, 123.7 | 90.5, 122.9 | 90.5, 122.5 | 90.6, 122.8 | 90.6, 122.7 | 90.5, 122.7 |
| Resolution (Å)^c^ | 50.00-2.00 | 50.0-1.79 | 50.0-1.70 | 50.0-1.77 | 50.0-1.66 | 50.0-1.75 |
|  | (2.07-2.00) | (1.82-1.79) | (1.73-1.70) | (1.80-1.77) | (1.69-1.66) | (1.78-1.75) |
| *R*_merge_ (%) | 9.7 (46.0) | 6.7 (58.8) | 6.0 (56.6) | 7.5 (57.2) | 6.1 (58.8) | 6.2 (51.6) |
| *I*/σ(*I*) | 10.4 (5.2) | 11.1 (4.1) | 13.0 (4.9) | 11.0 (5.5) | 12.5 (5.0) | 12.1 (4.3) |
| Completeness (%) | 99.3 (100.0) | 99.9 (100.0) | 100.0 (100.0) | 100.0 (100.0) | 100.0 (100.0) | 99.8 (99.8) |
| Redundancy | 6.0 (6.1) | 8.9 (8.7) | 10.6 (10.2) | 11.0 (11.0) | 11.0 (11.0) | 8.9 (8.6) |
| **Refinement** |  |  |  |  |  |  |
| Resolution (Å) | 50-2.0 | 50-1.79 | 50-1.70 | 50-1.77 | 50-1.66 | 50-1.75 |
| No. reflections | 37,505 | 52,514 | 60,851 | 54,460 | 65,790 | 56,028 |
| *R*_work_ / *R*_free_ (%) | 16.4 / 18.9 | 16.2 / 18.0 | 16.8 / 18.2 | 16.3 / 18.1 | 16.8 / 18.1 | 16.7 / 18.3 |
| Rmsd |  |  |  |  |  |  |
| Bond length (Å) | 0.013 | 0.010 | 0.009 | 0.010 | 0.009 | 0.010 |
| Bond angle (º ) | 1.548 | 1.414 | 1.376 | 1.359 | 1.321 | 1.372 |
| PDB ID | 5ZFB | 5ZF7 | 5ZF8 | 5ZF9 | 5ZF4 | 5ZFA |

^a^Data collected in beamline 17A at Photon Factory. ^b^Data collected in beamline 41XU at SPring-8. ^c^Values in parentheses are for the highest resolution shell.
