# Supplementary material for: Selective Cytotoxicity of Dihydroorotate Dehydrogenase Inhibitors to Human Cancer Cells Under Hypoxia and Nutrient-Deprived Conditions
Source: Front Pharmacol. 2018 Sep 4;9:997. doi: 10.3389/fphar.2018.00997 (PMC6131557; doi:10.3389/fphar.2018.00997)
Supplement: Supplementary file 2 [file Presentation_2.PPTX]

## Slide 1
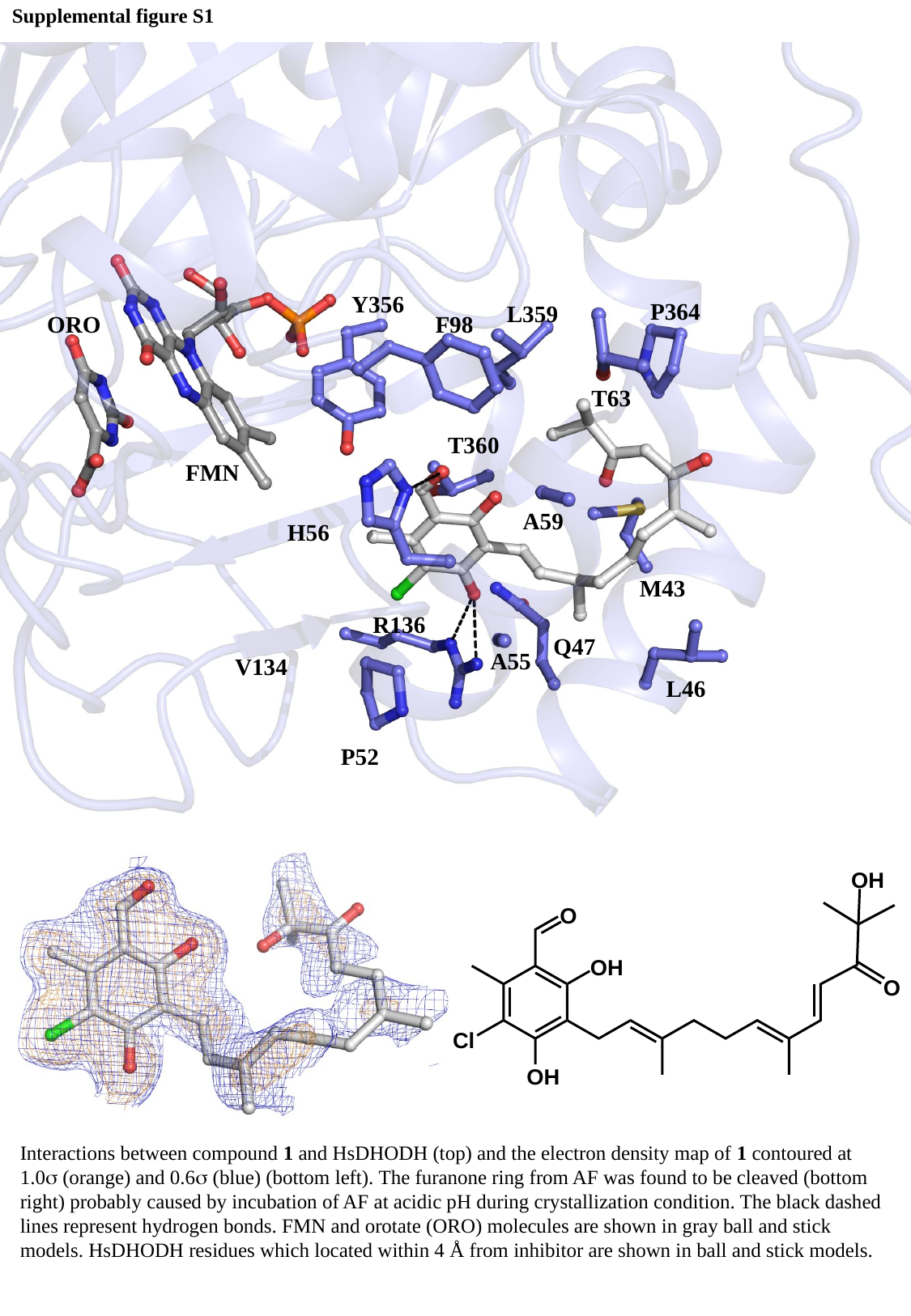

Supplemental figure S1
Y356
P364
L359
ORO
F98
T63
T360
FMN
A59
H56
M43
R136
Q47
A55
V134
L46
P52
Interactions between compound 1 and HsDHODH (top) and the electron density map of 1 contoured at 1.0 (orange) and 0.6 (blue) (bottom left). The furanone ring from AF was found to be cleaved (bottom right) probably caused by incubation of AF at acidic pH during crystallization condition. The black dashed lines represent hydrogen bonds. FMN and orotate (ORO) molecules are shown in gray ball and stick models. HsDHODH residues which located within 4 Å from inhibitor are shown in ball and stick models.

## Slide 2
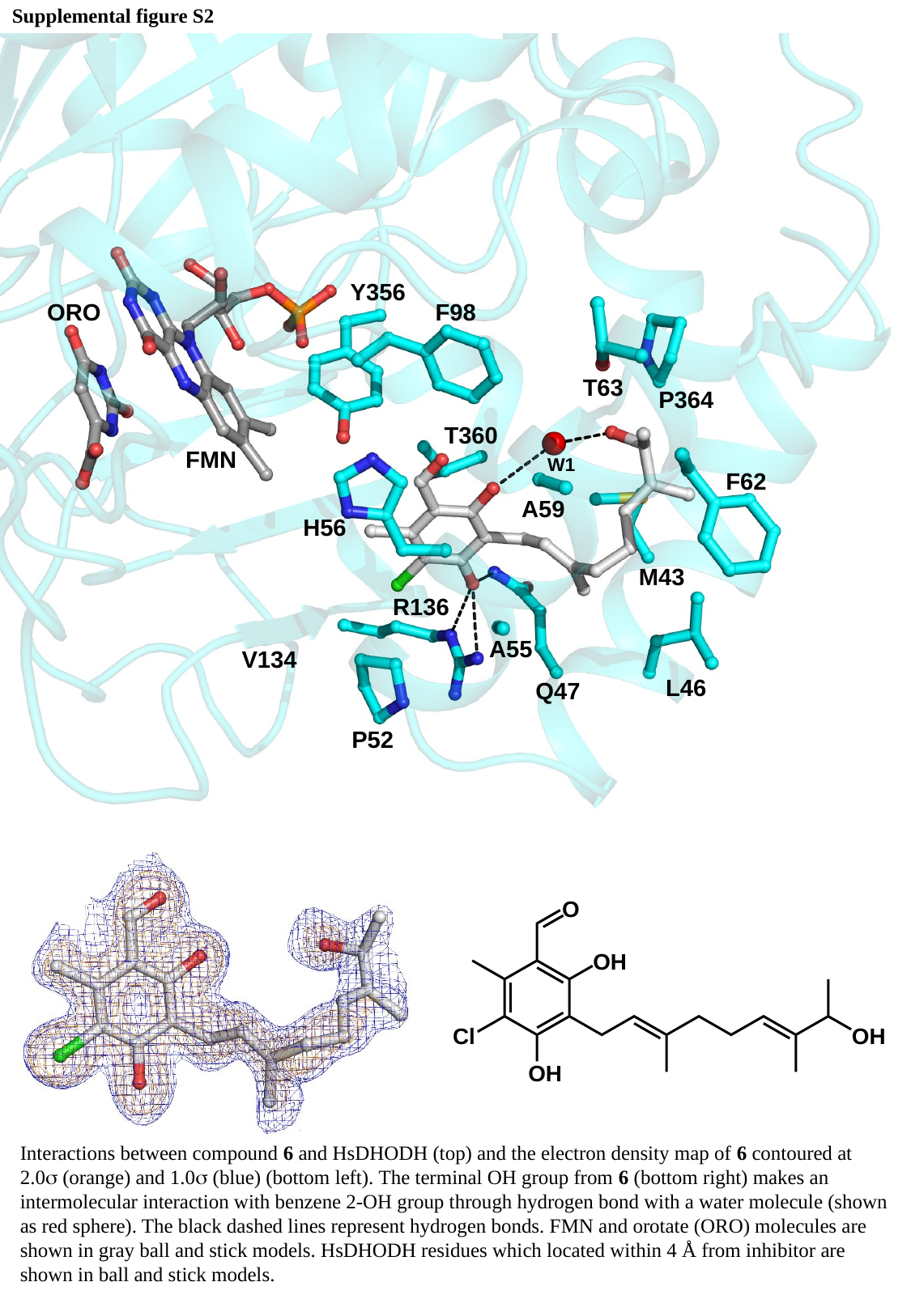

Supplemental figure S2
Y356
ORO
F98
T63
P364
T360
FMN
W1
F62
A59
H56
M43
R136
A55
V134
L46
Q47
P52
Interactions between compound 6 and HsDHODH (top) and the electron density map of 6 contoured at 2.0 (orange) and 1.0 (blue) (bottom left). The terminal OH group from 6 (bottom right) makes an intermolecular interaction with benzene 2-OH group through hydrogen bond with a water molecule (shown as red sphere). The black dashed lines represent hydrogen bonds. FMN and orotate (ORO) molecules are shown in gray ball and stick models. HsDHODH residues which located within 4 Å from inhibitor are shown in ball and stick models.

## Slide 3
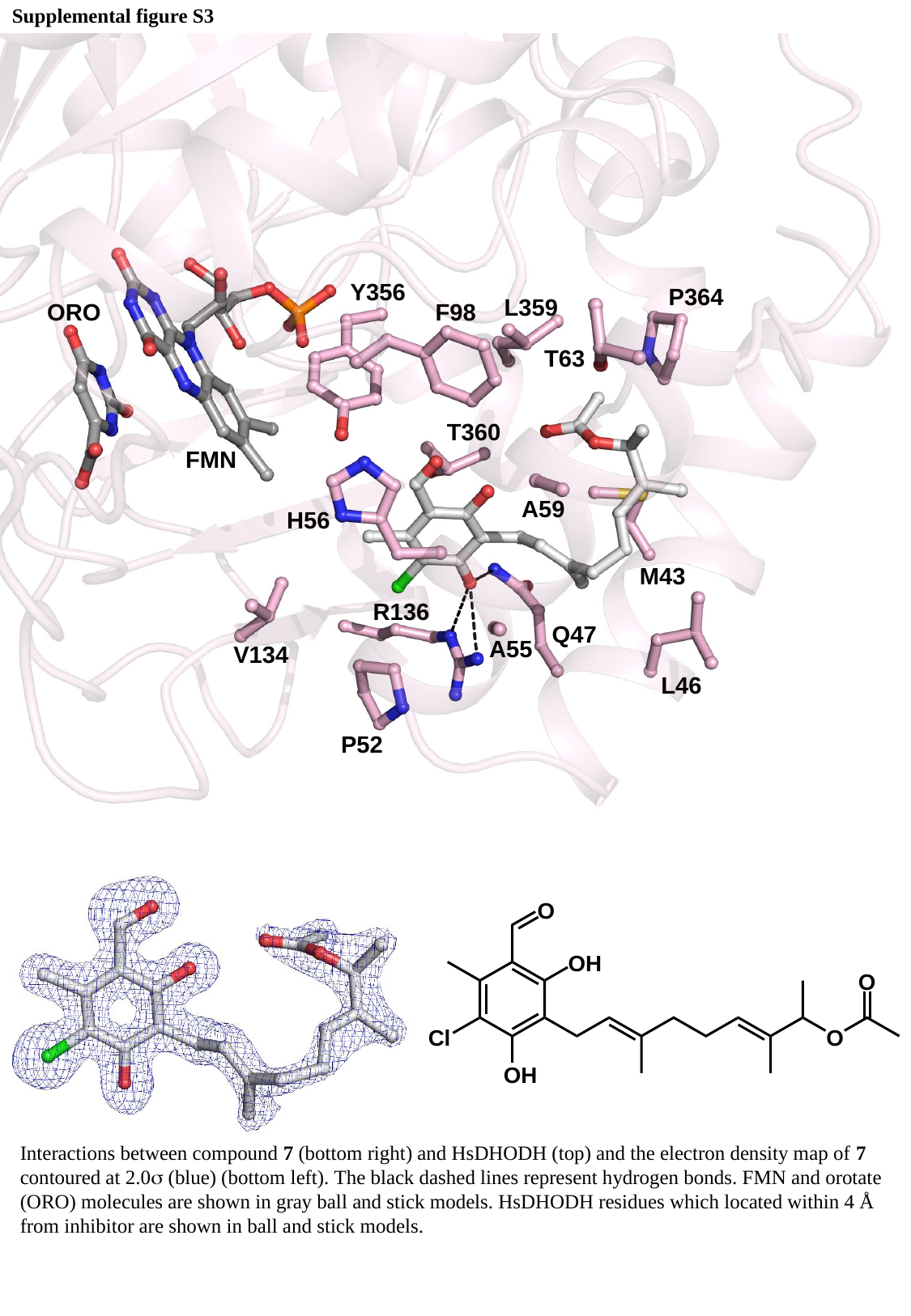

Supplemental figure S3
Y356
P364
L359
ORO
F98
T63
T360
FMN
A59
H56
M43
R136
Q47
A55
V134
L46
P52
Interactions between compound 7 (bottom right) and HsDHODH (top) and the electron density map of 7 contoured at 2.0 (blue) (bottom left). The black dashed lines represent hydrogen bonds. FMN and orotate (ORO) molecules are shown in gray ball and stick models. HsDHODH residues which located within 4 Å from inhibitor are shown in ball and stick models.

## Slide 4
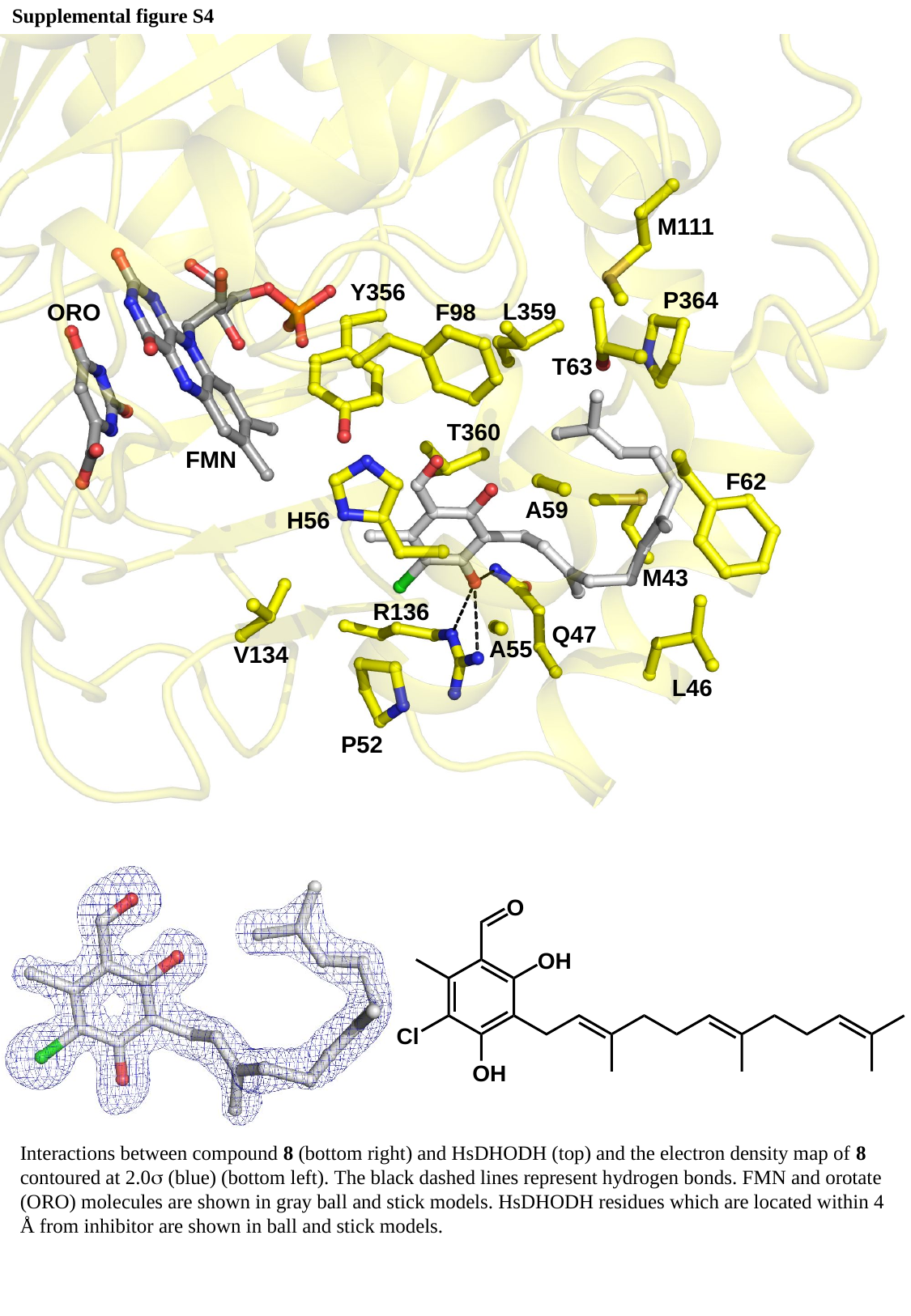

Supplemental figure S4
M111
Y356
P364
L359
ORO
F98
T63
T360
FMN
F62
A59
H56
M43
R136
Q47
A55
V134
L46
P52
Interactions between compound 8 (bottom right) and HsDHODH (top) and the electron density map of 8 contoured at 2.0 (blue) (bottom left). The black dashed lines represent hydrogen bonds. FMN and orotate (ORO) molecules are shown in gray ball and stick models. HsDHODH residues which are located within 4 Å from inhibitor are shown in ball and stick models.

## Slide 5
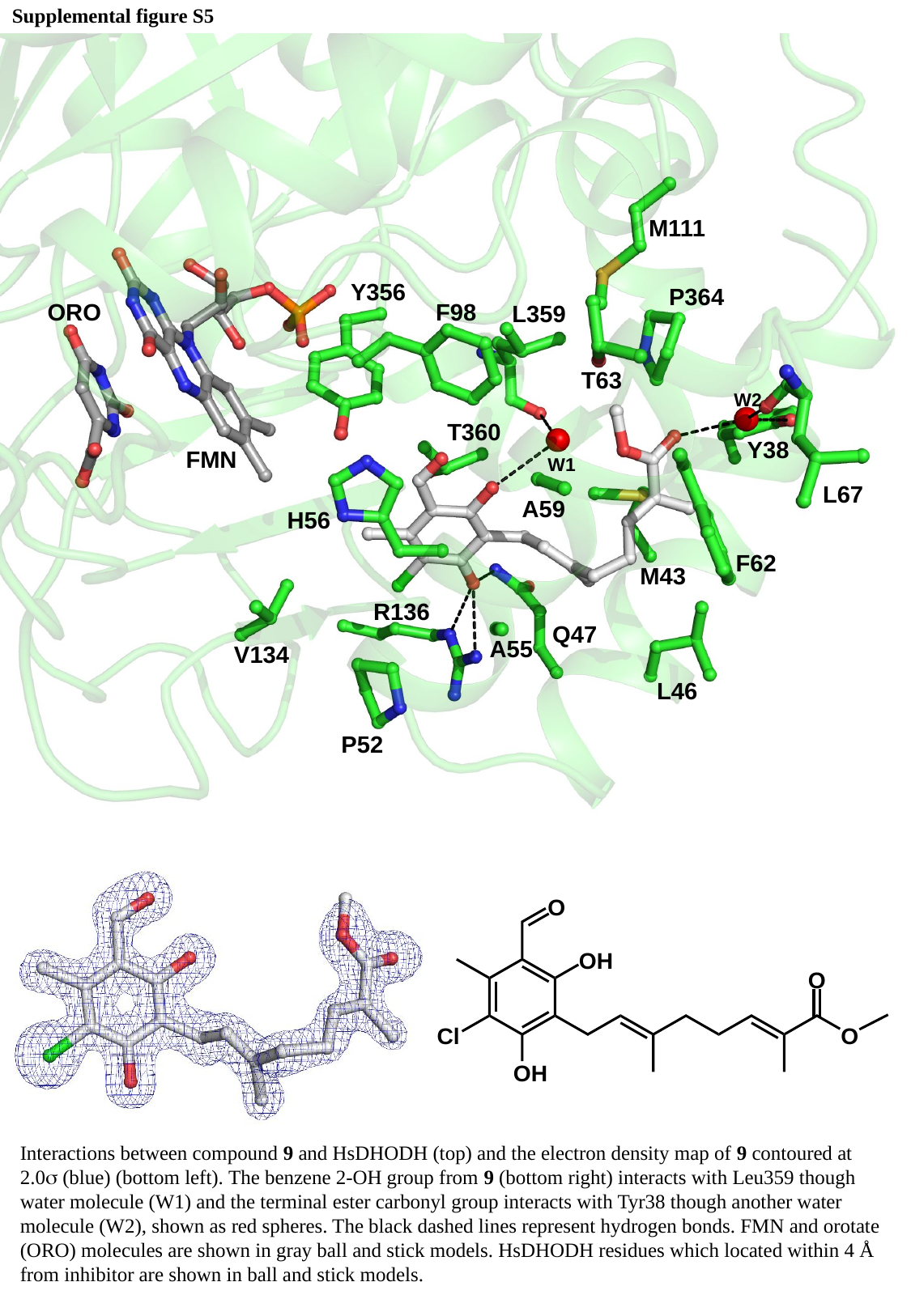

Supplemental figure S5
M111
Y356
P364
ORO
F98
L359
T63
W2
T360
Y38
FMN
W1
L67
A59
H56
F62
M43
R136
Q47
A55
V134
L46
P52
Interactions between compound 9 and HsDHODH (top) and the electron density map of 9 contoured at 2.0 (blue) (bottom left). The benzene 2-OH group from 9 (bottom right) interacts with Leu359 though water molecule (W1) and the terminal ester carbonyl group interacts with Tyr38 though another water molecule (W2), shown as red spheres. The black dashed lines represent hydrogen bonds. FMN and orotate (ORO) molecules are shown in gray ball and stick models. HsDHODH residues which located within 4 Å from inhibitor are shown in ball and stick models.

## Slide 6
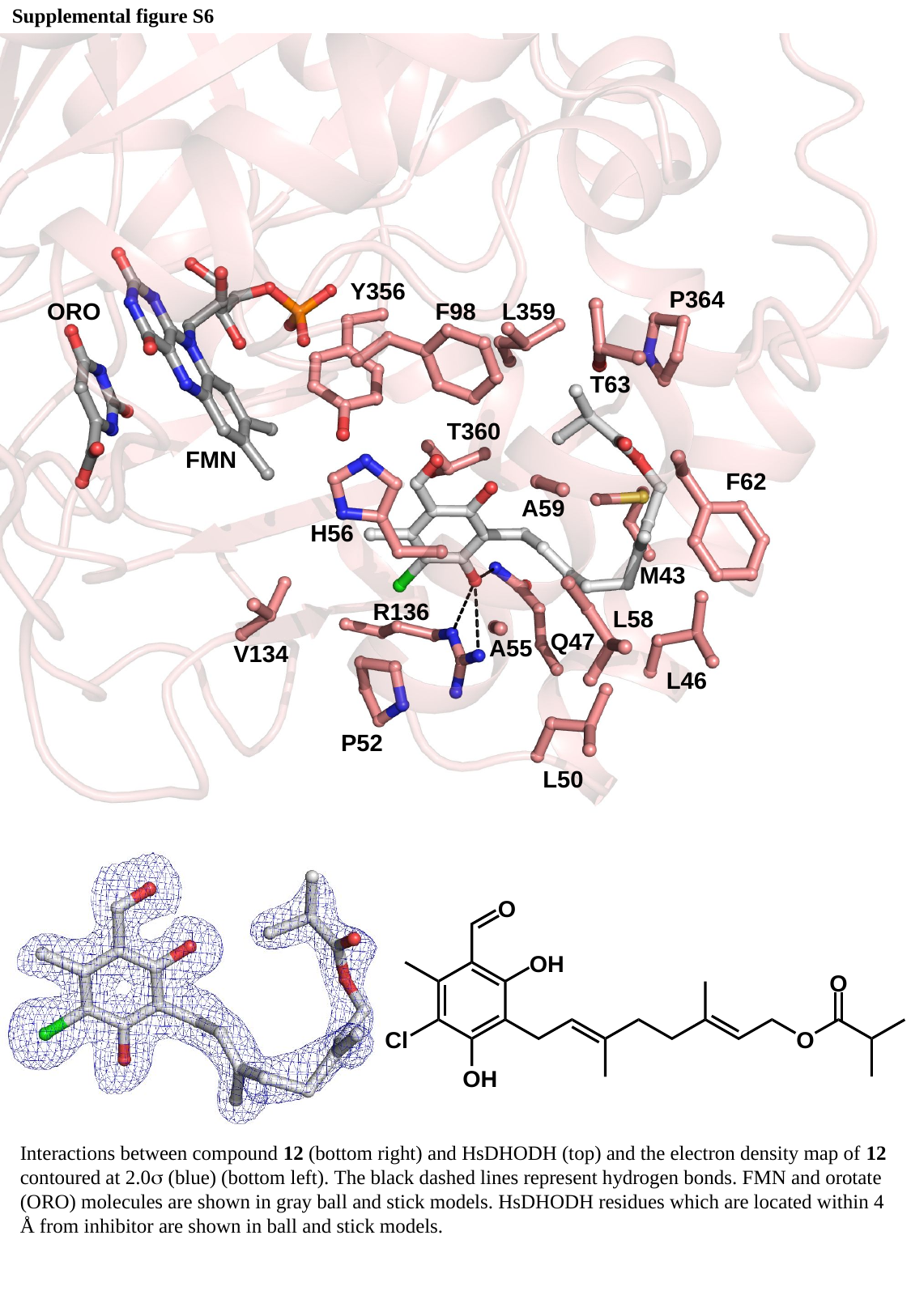

Supplemental figure S6
Y356
P364
ORO
L359
F98
T63
T360
FMN
F62
A59
H56
M43
R136
L58
Q47
A55
V134
L46
P52
L50
Interactions between compound 12 (bottom right) and HsDHODH (top) and the electron density map of 12 contoured at 2.0 (blue) (bottom left). The black dashed lines represent hydrogen bonds. FMN and orotate (ORO) molecules are shown in gray ball and stick models. HsDHODH residues which are located within 4 Å from inhibitor are shown in ball and stick models.

## Slide 7
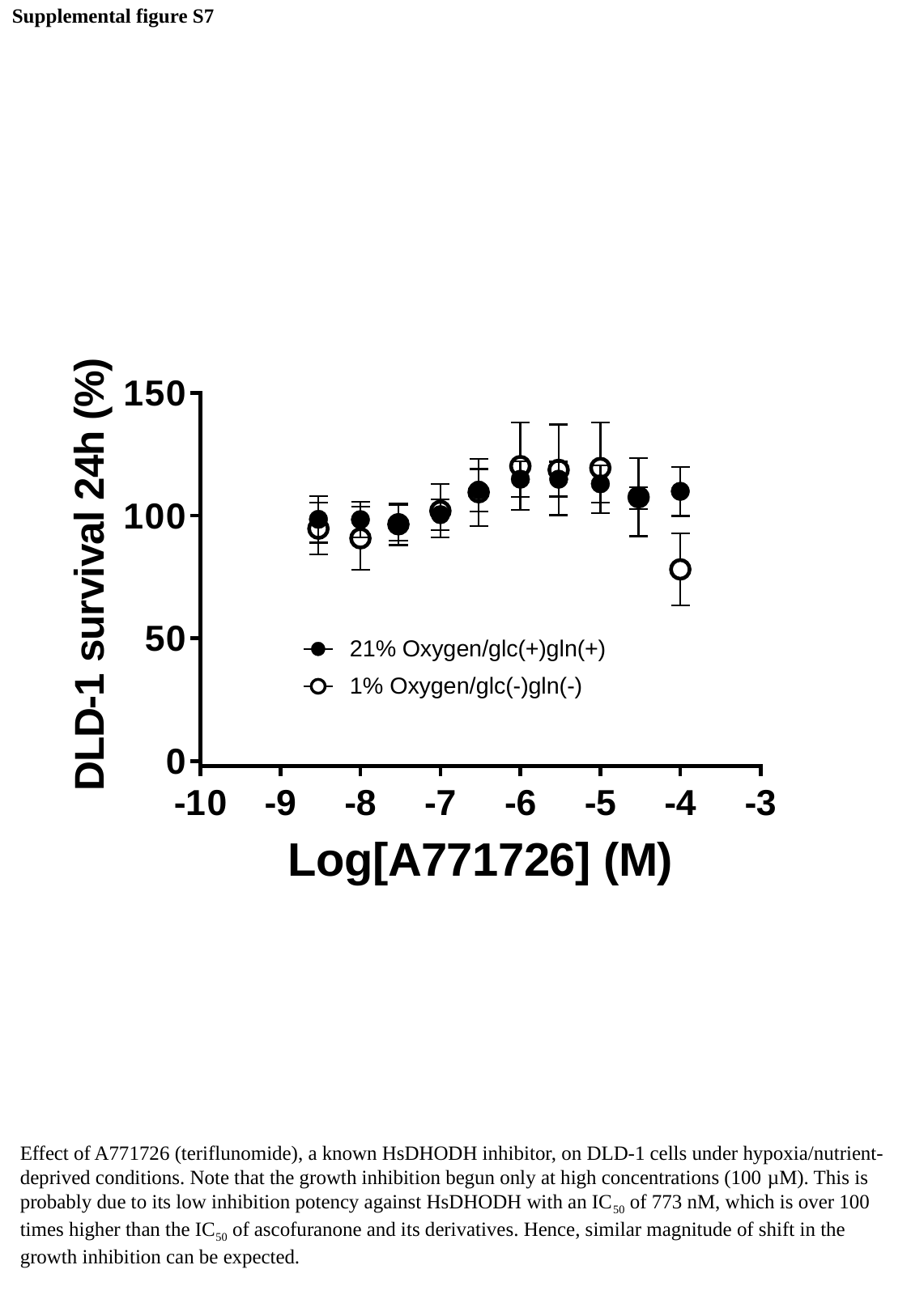

Supplemental figure S7
Effect of A771726 (teriflunomide), a known HsDHODH inhibitor, on DLD-1 cells under hypoxia/nutrient-deprived conditions. Note that the growth inhibition begun only at high concentrations (100 µM). This is probably due to its low inhibition potency against HsDHODH with an IC50 of 773 nM, which is over 100 times higher than the IC50 of ascofuranone and its derivatives. Hence, similar magnitude of shift in the growth inhibition can be expected.
